# Delivery of Short Interfering Ribonucleic Acid-Complexed Magnetic Nanoparticles in an Oscillating Field Occurs via Caveolae-Mediated Endocytosis

**DOI:** 10.1371/journal.pone.0051350

**Published:** 2012-12-07

**Authors:** Jenson Lim, Michael A. Clements, Jon Dobson

**Affiliations:** 1 nanoTherics Limited, Med IC4, Keele University Science and Business Park, Newcastle under Lyme, Staffordshire, United Kingdom; 2 School of Biosciences, University of Birmingham, Edgbaston, Birmingham, United Kingdom; 3 J. Crayton Pruitt Family Department of Biomedical Engineering, Department of Materials Science and Engineering, and Institute for Cell Engineering and Regenerative Medicine (ICERM), University of Florida, Gainesville, Florida, United States of America; 4 Institute for Science and Technology in Medicine, Keele University, Stoke-On-Trent, Staffordshire, United Kingdom; University of Queensland, Australia

## Abstract

Gene delivery technologies to introduce foreign genes into highly differentiated mammalian cells have improved significantly over the last few decades. Relatively new techniques such as magnetic nanoparticle-based gene transfection technology are showing great promise in terms of its high transfection efficiency and wide-ranging research applications. We have developed a novel gene delivery technique, which uses magnetic nanoparticles moving under the influence of an oscillating magnetic array. Herein we successfully introduced short interfering RNA (siRNA) against green fluorescent protein (GFP) or actin into stably-transfected GFP-HeLa cells or wild-type HeLa and rat aortic smooth muscle cells, respectively. This gene silencing technique occurred in a dose- and cell density- dependent manner, as reflected using fluorescence intensity and adhesion assays. Furthermore, using endocytosis inhibitors, we established that these magnetic nanoparticle-nucleic acid complexes, moving across the cell surface under the influence of an oscillating magnet array, enters into the cells via the caveolae-mediated endocytic pathway.

## Introduction

**Figure 1:**
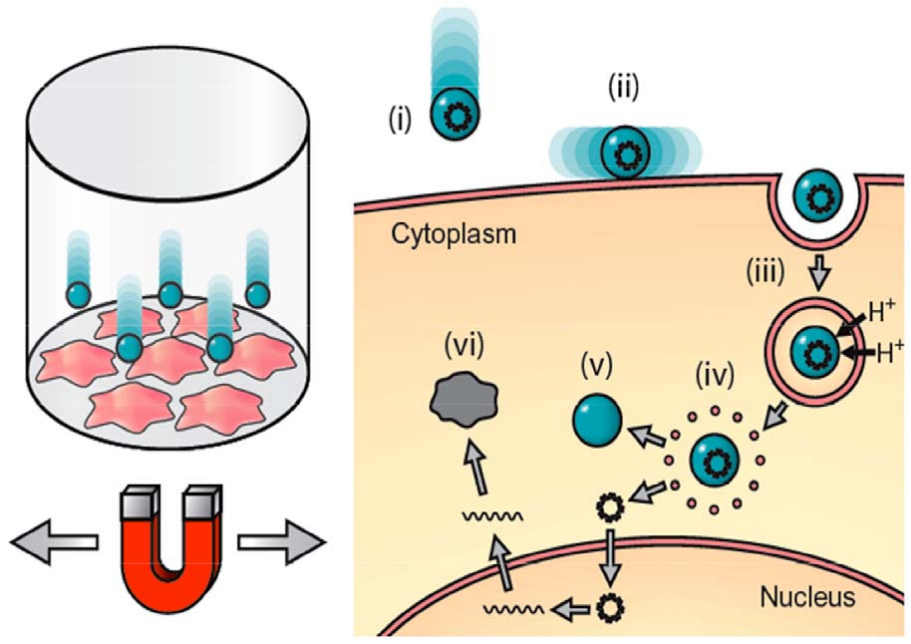
Principle of oscillating nanomagnetic transfection. Short interfering RNA (siRNA) or plasmid DNA is attached to magnetic nanoparticles and incubated with cells in culture (left). An oscillating magnet array below the surface of the cell culture plate pulls the particles into contact with the cell membrane (A) and drags the particles from side-to-side across the cells (B), mechanically stimulating endocytosis (C). Once the particle/nucleic acids complex is endocytosed, proton sponge effects rupture the endosome (D) releasing the nucleic acids (E) which either transcribes the target protein or silences the target genes (F) [3].

Recent decades have seen the rise of gene delivery technologies to introduce foreign genes into highly differentiated cells like neurons or leukocytes, as such cells are known to be resistant to either accepting or expressing exogenous genes. Such technologies range from the relatively inexpensive lipid-based (e.g Lipofectamine) or non-lipid based (e.g. Fugene) reagents to more costly nucleofection (e.g. Amaxa) or gene gun (e.g. Helios) methods (reviewed in [1]). Magnetic nanoparticle-based gene transfection technology is a relatively new and effective tool to introduce plasmid DNA or short interfering RNA (siRNA) into mammalian cells. Briefly, negatively-charged nucleic acids are electrostatically associated to positively-charged, polymer-coated superparamagnetic iron oxide nanoparticles (SPIONs). Next, these complexes are subjected to a strong high-gradient magnet field produced by arrays of permanent magnets sited underneath the cell culture plate. The effect of the field gradient is to essentially pull the particle/nucleic acid complex onto the surfaces of the cells. Our group has found that by introducing a linear oscillating motion to the magnet array, we can regulate the uptake of nanoparticle/plasmid DNA complexes (Figure 1).

Although we, and others, have shown successful transfection with this technology [2], [3], even with hard-to-transfect cells types such as mouse embryonic fibroblasts (MEF), human umbilical vein endothelial cells (HUVEC) [4], human osteosarcoma fibroblasts [5], primary rat oligodendrocyte precursor cells [6], purified primary rat astrocytes [7], primary cardiomyocytes (Subramanian et al, unpublished data) – with little negative effects on cell viability, migration, proliferation and differentiation, the potential of the technology is still to be further explored.

Remarkable differences were observed using human lung epithelial cells NCI-H292 transfected with a plasmid containing the luciferase reporter gene. A 2 Hz/0.2 mm frequency and amplitude of displacement of the oscillating magnet array showed higher transfection efficiency with little negative impact on cell viability compared with a static magnet system and two commercially available lipid-based reagents [2], [3]. Nanomagnetic transfection is also dependent on the magnet strength and its distance from the cell surface [3].

Here we show successful gene silencing of GFP and actin in stably-transfected GFP-HeLa cells and wild-type HeLa cells, respectively using this novel transfection system which outperformed a leading lipid reagent and a static magnet array system. Using endocytosis inhibitors, we also confirm that the route of entry for these nanoparticle-nucleic acid complexes is via the caveolae-mediated endocytic pathway, a process that appears to be enhanced by mechanical stimulation of the cells due to the oscillatory motion of the particle complexes across the cell surface.

## Methods

### Materials

Silencer GFP siRNA (siGFP) and the Negative Control (scrambled sequences, SCR) were purchased from Ambion/Invitrogen (Paisley, UK). Stealth siRNA against human Actin (siActin) was purchased from Invitrogen (Paisley, UK). Phosphate buffered saline, 24-well tissue cell culture plates and flasks (Costar) were purchased from Sigma (Dorset, UK). HeLa cells were purchased from ECACC/Sigma (Dorset, UK). Rat Aortic Smooth Muscle cells were a kind gift from Eva Pantazaka/Colin Taylor (University of Cambridge) [8]. Cells were maintained in the antibiotic-free medium consisting of high glucose MEM, 10% Fetal Bovine Serum (FBS) and 2 mM L-glutamine, purchased from Biosera (East Sussex, UK). Endocytosis inhibitors were purchased from either Calbiochem/Merck (Nottingham, UK) or Sigma (Dorset, UK).

### DNA Constructs

Eukaryotic expression vector pEGFP-N1 (CMV promoter driving gene encoding green fluorescence) was purchased from Clontech (Mountain View, USA). Plasmid DNA was prepared using the Qiagen EndoFree Plasmid Purification kit (Qiagen, Crawley, UK), and maintained in endonuclease-free water (Sigma, Dorset, UK) at −80°C.

### Creation of Stably Transfected GFP-HeLa

90000 HeLa cells per well were seeded onto a 24-well tissue culture plate and left overnight in a 37°C, 5% CO_2_ incubator. 0.6 µg of pEGFP-N1 (Clontech, UK) was complexed with 0.6 µl of nTMag (nanoTherics, Stoke-On-Trent, UK) in serum-free MEM for 15 min before transferring to the wells containing HeLa cells. nTMag is Fe_3_O_4_ dispersed in a polyethylenimine-HCl matrix; zeta potential: +23.4 mV; particle size distribution: 1.8 (polydisperse index). Cell were transfected using the magnefect-nano II system (nanoTherics, UK) before transferring the 24-well plate to the incubator for 48 hr. Fresh medium was replaced containing 0.5 mg/ml G418 (Biosera, UK). After 14 days, brightest GFP-expressing colonies of HeLa cells were selected visually and transferred to a 96-well plate. Another round of selection was conducted after a further 20 days, with cells then transferred to a 6-well plate. Finally GFP-HeLa cells were expanded to a 25 cm^2^ flask and cell sorted (Flow Cytometry Core Service, University of Sheffield) to enrich the population of GFP-expressing HeLa cells. Approximately 1 million GFP-HeLa cells were frozen down (liquid nitrogen) in a mixture containing 10% DMSO/FBS.

### Transfection of Cells

#### Oscillating nanomagnetic transfection

10,000/50,000 RASMC, 50,000 HeLa or 100,000 GFP-HeLa in antibiotic-free medium were seeded in 24-well plates, incubated at 37°C, 5% CO_2_, for 24 hr before addition of transfection complexes. To prepare transfection complexes, 10–300 nM siActin or 0.1–20 nM siGFP were diluted in 100 µl serum-free media, added to 1.2–2.0 µl nTMag (nanoTherics, UK) and mixed. After 20 min, complexes were added drop-wise to cells, with control wells comprising untransfected cells. Plates were incubated in the presence of an oscillating magnetic field (2 Hz at 0.2 mm amplitude of displacement) using the magnefect-nano II (nanoTherics, UK) system.

#### Lipofectamine

Cells were seeded into 24-well tissue culture plates as described before. Lipofectamine 2000 complexes were prepared in serum free medium using 33 nM of siRNA with 1 µl of Lipofectamine 2000 per well following the manufacturer’s recommended protocol. After 20 min incubation, complexes were added drop-wise to cells, and incubated at 37°C, 5% CO_2_, before analysis.

#### Nucleofection

Cells were transfected by nucleofection (Amaxa/Lonza, DE) using program I-13, according to manufacturer’s instructions. Cells were left to express plasmids for 72 or 168 hr before flow cytometric analysis.

### Flow Cytometry

Transfected cells were washed in 0.5% bovine serum albumin and phosphate-buffered saline (PBS) and analysed for the relative fluorescence of gated cells, using a FACSort analyser (Becton Dickinson, USA). Median fluorescence intensity of gated cells was determined through the FL1 channel.

### Viability Assay

Cell viability was assessed using the MTT [3-(4,5-dimethylthiazol-2-yl)-2,5-diphenyltetrazolium bromide] assay, which is based on the ability of a mitochondrial dehydrogenase enzyme from viable cells to cleave the tetrazolium rings of the pale yellow MTT to form dark blue formazan crystals. 0.5 mg/ml of MTT dissolved in serum-free medium was added to cells in wells. Cells were allowed to accumulate crystals for 2–4 hr in a 37°C, 5% CO_2_ incubator, before they were solubilised with dimethyl sulfoxide and read using spectrophotometer at a wavelength of 595 nm. Number of viable cells was directly proportional to the level of the formazan product created [9], [10].

### Adhesion Assay

This protocol was based on Dormoy-Raclet et al [11]. Six-well plates were coated with 0.1% gelatin at 4°C overnight. To avoid nonspecific binding, wells were blocked with 1% bovine serum albumin (BSA) for 1 hr at 37°C. Control, siRNA-transfected HeLa cells were trypsinised and plated on the matrix-coated wells for 6 hr. Adherent cells were fixed with methanol at −20°C and stained with Hoechst 33342 (Sigma, UK). The nuclei were counted by fluorescence microscopy (IX71, Olympus, UK). A minimum of three images were captured at fixed exposure settings, and an average of the number of nuclei from cells transfected with target siRNA was related to a scrambled control. Significance was tested using Student’s *t* test. P<0.05 was considered significant and is indicated by a single asterisk.

### Treatment with Inhibitors

Cells were treated with 2–50 µg/ml chlorpromazine (CHL), 1.0–25 µg/ml filipin, 0.2–5 µM 5-(N-ethyl-N-isopropyl) amiloride (EIPA) (Sigma, Dorset, UK), 40–1000 µM genistein (GENI) or 8–200 µM dynasore (DYNA) (Calbiochem/Merck, Nottingham, UK) in serum-free culture medium for 30 min at 37°C. Subsequently, nTMag complexed with GFP in serum-free medium were added drop-wise to cells, transfected at 2Hz with 0.2 mm amplitude of displacement using the magnefect-nano II system (nanoTherics, UK) for 30 min, and incubated for a further 1 hr (2 hr total contact time with inhibitors). Fresh serum-containing medium was replaced and cells were analysed for transfection efficiency and viability using fluorescence microscopy and the MTT assay after 24 hr, respectively. Total transfection was obtained by multiplying the proportion of GFP transfected cells by its corresponding %viability.

## Results

### Knock-down of GFP from Stably Transfected GFP-HeLa using an Oscillating Magnet Array

**Figure 2:**
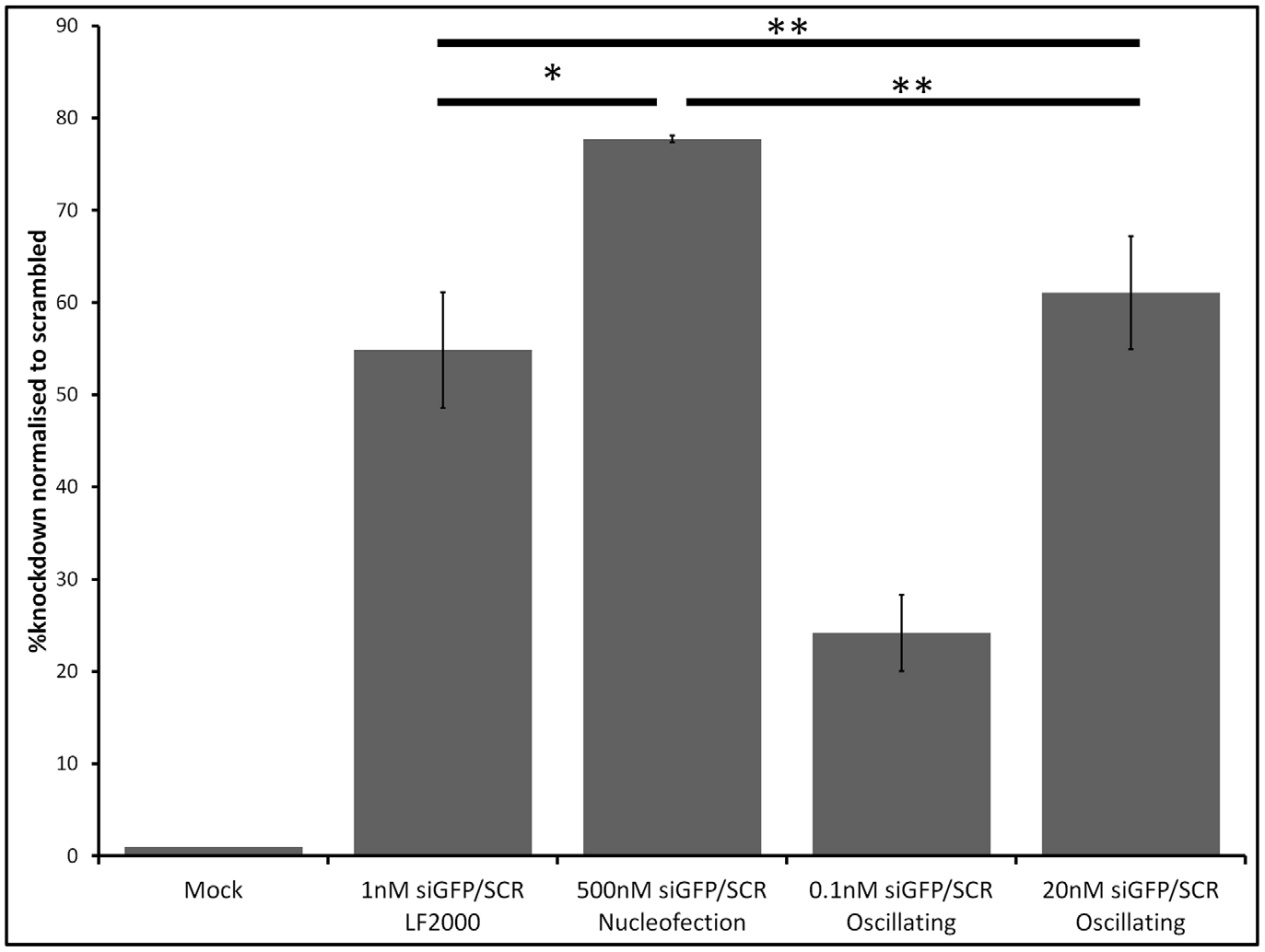
Knockdown of GFP from GFP-HeLa cells. Stably-expressing GFP-HeLa cells were transfected, either with a lipid reagent, nucleofection method or magnetofection using an oscillating magnet (2Hz frequency amplitude and 0.2 mm displacement), as indicated. Magnetofection using nTMag only was set as negative control (Mock). Fluorescent intensity (Median) was determined 72 hr post-transfection and knockdown levels were normalised to a scrambled control. Results are expressed as the mean±SD of at least three independent experiments. *, p<0.05; **, p≥0.05.

We sought to transfect short interfering ribonucleic acid (siRNA)-associated magnetic nanoparticles into mammalian cells using the oscillating magnet array. For simplicity, and as proof of principle, HeLa cells were first stably transfected with a plasmid encoding green fluorescence protein (GFP), selected (G418) and sorted on the basis of GFP as described in the Methods section. Stably-expressing GFP-HeLa cells were transfected with siRNA against GFP using Lipofectamine 2000, nucleofection technology (both according to manufacturers’ recommended instructions) and the oscillating magnet array system (Figure 2).

Gene silencing was determined using fluorescence median within a gated population of cells and normalising to a scrambled siRNA control. Although the nucleofection method was able to silence GFP after 72 hr (77.7±0.4%) using a high dose of siRNA (500 nM), this was not sustained as observed after 168 hr (43.6±3.8%, data not shown). The oscillating magnet array system, with a lower dose of siRNA (20 nM), showed approximately 61.1±6.1% knockdown of GFP, both after 72 hr and 168 hr (Figure 2 and data not shown). Cells transfected using Lipofectamine 2000, in accordance with manufacturer’s instructions, demonstrated good gene silencing (54.8±6.3%), though cell rounding was observed which correlated with reduced viability (data not shown). These results demonstrate the ability of the oscillating magnet array system to achieve gene knock-down in a cell line with an over-expressed reporter gene.

### Gene Silencing of Actin from HeLa and Rat Aortic Smooth Muscle Cells

Next we wanted to silence an endogenous protein using the oscillating magnet array technology. Actin, a major cytoskeletal protein, found ubiquitously in eukaryotes, is known to be involved in a range of cellular activities including adhesion [12]. Recent work has demonstrated that transfecting 120 nM siRNA against β-actin in HeLa cells using Lipofectamine Plus led to approximately 60% reduction of reattached HeLa cells after 72 hr, as observed using adhesion assays, when compared to the scrambled control siRNA [11]. We looked specifically at the gene silencing by transfection using magnetic nanoparticles complexed with siRNA against β-actin and the oscillating magnet array, and to determine its effects using a physiological assay.

**Figure 3:**
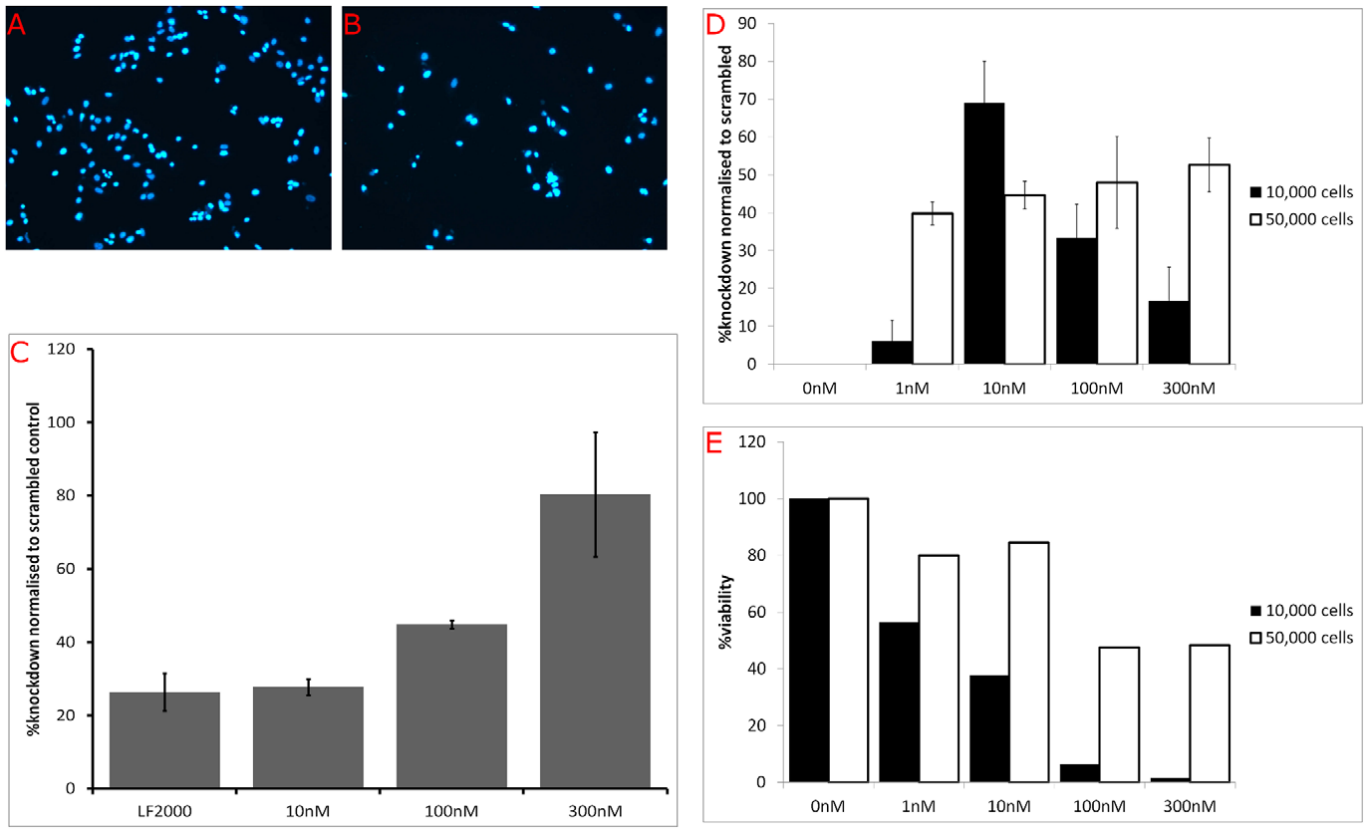
Knockdown of Actin from HeLa and Rat Aortic Smooth Muscle Cells. HeLa (A–C) or rat aortic smooth muscle cells (D, E) transfected with the either scrambled siRNA or siRNA against actin, as indicated, were seeded on six-well gelatine-coated plates 72 hrs post-transfection. Adherent, Hoechst–stained (nuclear) HeLa cells after transfection with scrambled siRNA (A) or actin siRNA (B) were counted by fluorescence microscopy, as described in the Methods section. Knockdown of actin in HeLa (C) or rat aortic smooth muscle cells (D) and its viability (E) was expressed as a percentage of the number of adherent cells transfected by actin siRNA over its scrambled control. Data represent the mean±SD of at least three independent experiments.

In HeLa cells, we were successful in introducing siRNA against β-actin using the oscillating magnet array, as evidenced by a decrease in reattached HeLa cells onto gelatine-coated plates 72 hr after transfection [11] (c.f. Figure 3A and 3B). This occurs in a (siRNA) dose-dependent manner, whereas cells transfected using Lipofectamine 2000 (with 33 nM of siRNA in accordance to the manufacturer’s instruction) showed low reattached HeLa cells (Figure 3C). At a higher dose of siRNA against actin (300 nM), the effect was maintained over time (data not shown).

A similar effect can be seen in a primary cell type, the rat aortic smooth muscle cells (Figure 3D and 3E). The actin gene was knocked-down at varying levels, in a dose- and cell density- dependent manner. At 10,000 cells per well, the highest knock down of actin was using 10 nM of siRNAs (69.0±11.0%, as determined using the adhesion assay, normalised to scrambled control). Decreasing viability was seen with increasing dose of complexes, suggesting some toxic effects of the complexes at higher concentrations. Despite the very low viabilities, it must be noted that the MTT viability assay, as a colorimetric assay, is affected by sensitivity issues. The readout is probably an underestimation as to the actual number of cells remaining, based on a manual count of nuclei after transferring cells from one plate to another.

This contrasted with wells plated with 50,000 cells, which had more consistent levels of knock-down at all concentrations of siRNAs and viabilities (between 39.8±3.0% for 1 nM to 52.6±7.1% for 300 nM; Figure 3D) presumably due to ‘diluting’ effect of complexes by the higher number of cells. Unsurprisingly, transfection efficiencies also impacted cell viabilities with transfection using 10,000 cells per well showing a clear dose-dependent effect, whereas, transfection with 50,000 per well was better in maintaining high cell viabilities. Overall, these results demonstrate success in delivering siRNA into HeLa cells using magnetic nanoparticles and the accompanying oscillating magnet array and its subsequent gene silencing effects.

### Nucleic Acid-nanoparticle Complexes Enter HeLa Cells in a Caveolae-dependent Endocytosis

Finally, in order to understand the mechanism by which these nucleic acid-nanoparticle complexes enter into the target cells, we investigated the impact of endocytosis blockers on nanomagnetic transfection efficiency. It has been reported that non-viral gene carriers including magnetic nanoparticles, are energy dependent and exploit the endocytic pathway to gain entry into their target cells [13], [14], [15]. Hence we used varying doses of endocytosis inhibitors such as 5-(*N*-ethyl-*N*-isopropyl) amiloride (EIPA, a macropinocytosis blocker) [16], [17], Chlorpromazine (a drug that disrupts clathrin-mediated endocytosis) [18], [19], Dynasore (a dynamin inhibitor – dynamin promotes the formation of clathrin-coated vesicles, a crucial stage in clathrin-mediated endocytosis) [20], [21], [22], Genistein and Filipin (a tyrosine kinase inhibitor that inhibits the caveolae-mediated endocytosis) [18], [23] to determine the pathway by which the nucleic acid-nanoparticle complexes enter into HeLa cells. In order to assay the effect of these blockers on transfection, we complexed nTMag with pEGFP-N1 plasmid (which over expresses Green Fluorescent Protein) to report on the entry of the magnetic nanoparticles.

**Figure 4:**
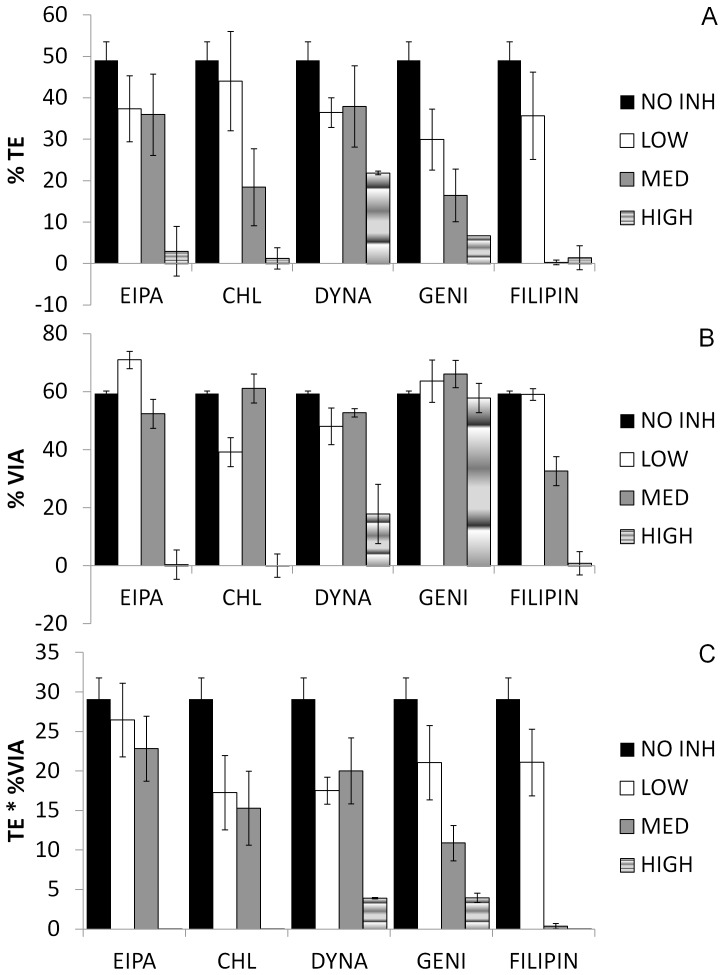
Effects of inhibitors on transfection and cell viability. HeLa cells were transfected with pEGFP-N1 in the presence of varying doses of inhibitors, as indicated and described in the Methods section. Transfection efficiency (A), viability (B) and Total Transfection (C) were determined 24 hr post-transfection using flow cytometry or MTT assay, respectively, and expressed as the mean±SD of at least three independent experiments.

On analysis 24 hrs post transfection, we found transfection of HeLa cells with nTMag associated with pEGFP-N1 plasmid was affected by low doses (Figure 4A, white bars) of endocytosis inhibitors (all p<0.05) when compared to the untreated control (Figure 4A, black bars, 49.0±4.5%). Unsurprisingly, cell viabilities were minimally affected with low doses of all endocytic inhibitors. Medium doses (optimal concentrations, based on abovementioned publications; grey bars) of Genistein (16.5±6.4%) and Chlorpromazine (18.5±9.3%) affected transfection efficiencies, with little effect on cell viabilities (Figure 4B,C). Filipin completely inhibited transfection of HeLa cells with some decrease in cell viability (32.7±5.0%, p<0.05 c.f. no inhibitor control) while transfection efficiencies remained virtually unchanged for Dynasore and EIPA. High doses (hatched bars) of EIPA, Chlorpromazine and Filipin decreased both transfection efficiencies and cell viabilities. High doses of Dynasore demonstrated a modest decrease in transfection efficiency (21.9±0.5%), though it did not inhibit viability (17.9±10.3%). Interestingly, high doses of Genistein had no effect on cell viability, although it reduced transfection efficiency dramatically (6.7±0.3%). Therefore, we suggest that these plasmid-associated nanoparticles enter cells via the caveolae-mediated pathway.

## Discussion

Previously we have demonstrated the use of a horizontal/lateral motion to a magnet array improves the transfection of both easy- and hard-to-transfect cell lines (as described earlier). In this study we explored the potential of this technique for gene silencing using short-interfering RNA (siRNA) and to understand the molecular mechanism behind magnetofection using an oscillating magnet array. As proof of principle, we created stably expressed green fluorescence protein (GFP) in HeLa and HEK293 cells by transfecting using our oscillating magnet array system and selecting using G418 antibiotics. Following sorting based on GFP fluorescence, we succeeded in silencing the GFP gene using siRNA complexed with nTMag and transfected with our oscillating magnet array. Our oscillating magnet array system had the benefit of using less siRNA in order to achieve high levels of gene silencing, compared with the nucleofection method. Also our system appears to prolong siRNA knock-down function without the overgrowth of untransfected cells or cell death over time, something we observed with the nucleofection and lipid reagent methods, respectively.

Next we examined knock-down of an endogenous gene. Actin, a major cytoskeletal protein, found ubiquitously in eukaryotes, is known to be involved in cell division, phago-/endo-/exo-cytosis, motility/adhesion, etc [12], [24], [25], [26]. Transfecting siRNA against β-actin in HeLa cells led to a decrease in cell growth and strong cellular blebbing [27] or a marked decrease in stress fibre formation, cell adhesion and migration [11]. We observed a physiological knock-down of actin in HeLa cells, in a dose-dependent manner, as observed using the adhesion assay. Others have successfully knocked-down various genes in HeLa cells or other cell lines using a combination of cationic liposomes and/or magnetofection with a stationary magnet [28], [29], [30]. While transfection efficiencies of siRNA between lipid methodology and magnetofection were similar, the significant difference is one of viability – with lipid methodology causing greater cell death compared to magnetofection [31]. We successfully silenced actin in primary rat aortic smooth muscle cells and demonstrated that this occurs in a cell density- and dose- (i.e. siRNA-nanoparticle complexes) dependent manner.

Another aim of this work was to determine route of entry of the magnetic nanoparticle complexes into the cell. Magnetofection of HeLa cells involves a combination of non-specific, clathrin- and caveolae- dependent endocytosis, as determined using antimycin A, a non-specific endocytic and other energy-dependent processes [32] and markers specific for clathrin- (FITC-labelled transferring-polylysine) and caveolae- (Alexa Fluor 594 cholera toxin subunit B) mediated endocytosis [33]. More recently others have demonstrated that in the absence of a magnet, magnetite-coated polystyrene particles (300 nm diameter) were taken up by astrocytes in a temperature dependent manner, suggesting the need for energy during uptake. Blocking studies showed that macropinocytosis (as demonstrated using amiloride and EIPA), not caveolin-dependent endocytosis, was involved with the uptake of such magnetic nanoparticles by astrocytes. Clathrin-dependent endocytosis was only partially involved [13].

Previously we have shown that using our oscillating magnet array (set to oscillate at 2Hz with a 0.2 mm displacement), NCI-H292 cells transfected with magnetic nanoparticles associated with a plasmid containing a luciferase reporter had 50% decrease in luciferase expression, in the presence of either antimycin A (1 µg/ml) or nystatin (25 µg/ml), in general agreement with [33] (data not shown). However, antimycin A is a metabolic inhibitor and blocks most energy-dependent processes and nystatin, a cholesterol depleting compound which disrupts caveolae/lipid rafts and destabilises clathrin coated pits as well [34], [35] and moreover it was shown that nystatin switches the internalization of endostatin from caveolae/lipid rafts to clathrin-coated pits in HUVECs [36]. Therefore there is a need to ascertain the route of entry for the magnetic nanoparticle complexes when under the influence of an oscillating magnet array.

Using a combination of endocytosis inhibitors, we found that magnetic nanoparticles complexed with plasmid encoding GFP, entered HeLa cells via caveolae-mediated endocytosis. While our results are largely in agreement with other groups [33], there are some differences. In [33], they found that in three different cell lines (HeLa included), magnetic nanoparticles coated with either a fluorescently-labelled, clathrin- or caveolae- specific marker, entered the cells via both pathways as determined using fluorescence microscopy. This slight discrepancy could be due to either our use of smaller nanoparticles (100 nm c.f. 200 nm in [33]), our use of the oscillating magnet array which could favour one pathway over the other due to mechanical stimulation of the cells, or our use of endocytosis inhibitors for this study.

While we are aware of the pitfalls of using pharmacological inhibitors to study the endocytic route of entry for the magnetic nanoparticles – e.g. lack of specificity on the part of the inhibitor itself, efficacy of inhibition and viability of cells after exposure to inhibitors and transfection is highly cell dependent [14], we chose endocytosis inhibitors based on current knowledge as to their specificity and the impact they have on the pathway in question [14]. Overall, while there is more work that needs to be undertaken to further confirm these results, these preliminary results indicate that using our oscillating magnet array system, magnetic nanoparticles enter cells via a caveolae-mediated pathway.
